# Combining neuropsychological assessment and structural neuroimaging to identify early Alzheimer's disease in a memory clinic cohort

**DOI:** 10.1002/brb3.3505

**Published:** 2024-04-30

**Authors:** Yi‐En Quek, Yi Leng Fung, Pierrick Bourgeat, Simon J. Vogrin, Steven J. Collins, Stephen C. Bowden

**Affiliations:** ^1^ Melbourne School of Psychological Sciences The University of Melbourne Parkville Victoria Australia; ^2^ The Australian e‐Health Research Centre CSIRO Health and Biosecurity Herston Queensland Australia; ^3^ Department of Clinical Neurosciences St. Vincent's Hospital Melbourne Fitzroy Victoria Australia; ^4^ Department of Medicine The Royal Melbourne Hospital The University of Melbourne Parkville Victoria Australia

**Keywords:** Alzheimer's disease, magnetic resonance imaging, memory clinic, neuropsychology

## Abstract

**Introduction:**

The current study examined the contributions of comprehensive neuropsychological assessment and volumetric assessment of selected mesial temporal subregions on structural magnetic resonance imaging (MRI) to identify patients with amnestic mild cognitive impairment (aMCI) and mild probable Alzheimer's disease (AD) dementia in a memory clinic cohort.

**Methods:**

Comprehensive neuropsychological assessment and automated entorhinal, transentorhinal, and hippocampal volume measurements were conducted in 40 healthy controls, 38 patients with subjective memory symptoms, 16 patients with aMCI, 16 patients with mild probable AD dementia. Multinomial logistic regression was used to compare the neuropsychological and MRI measures.

**Results:**

Combining the neuropsychological and MRI measures improved group membership prediction over the MRI measures alone but did not improve group membership prediction over the neuropsychological measures alone.

**Conclusion:**

Comprehensive neuropsychological assessment was an important tool to evaluate cognitive impairment. The mesial temporal volumetric MRI measures contributed no diagnostic value over and above the determinations made through neuropsychological assessment.

## Graphical abstract

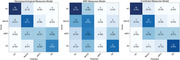

## 1 INTRODUCTION

The early, objective identification of cognitive impairment occurring in Alzheimer's disease (AD), such as that presenting in its prodromal stage, amnestic mild cognitive impairment (aMCI), plays a fundamental role in symptom clarification and prognostication for optimal patient care (Dubois et al., 2016). However, the diagnosis of aMCI or other, nonamnestic forms of MCI can be challenging because MCI presents with only mild changes in cognition and is an etiologically heterogeneous syndrome (Edmonds et al., 2016). Hence, assessment methods that can aid in the identification of individuals with MCI and offer prognostic insight are likely to be clinically useful.

In the clinical evaluation of patients suspected to have MCI, cognitive testing represents an important tool for the objective confirmation of cognitive impairment (Albert et al., 2011; Jacova et al., 2007). As there is some degree of measurement error inherent in any cognitive test, single test scores may not be sufficiently precise to detect mild changes in cognition (Haxby et al., 1992; Klekociuk et al., 2016). Composite scores that are derived from test batteries reduce the influence of the error of its individual components, providing improved precision over a single test score (Haxby et al., 1992; Jonaitis et al., 2019). Composite scores are, therefore, likely to be more reliably sensitive to the mild cognitive changes in MCI.

In the clinical setting, structural magnetic resonance imaging (MRI) is well positioned to contribute to the objective assessment of cognitive dysfunction, especially when due to a neurodegenerative process, as it is widely available, noninvasive, and relatively inexpensive. MRI has demonstrated sensitivity to the earliest structural brain changes in AD. In particular, longitudinal MRI studies have shown that disease‐related atrophy begins to occur in the entorhinal and transentorhinal cortices approximately 10 years prior to a diagnosis of MCI and in the hippocampus approximately 2−4 years prior to a diagnosis of MCI (Kulason et al., 2020; Younes et al., 2014). These findings suggest that MRI‐based measures of the entorhinal and transentorhinal cortices and hippocampus may serve as biomarkers for the early stages of AD.

Guidelines for the clinical assessment of MCI have proposed that supplementing the clinical evaluation with biomarker testing may enhance diagnostic accuracy (Albert et al., 2011; Petersen, 2004). Therefore, combining neuropsychological evaluation with MRI measures may yield improved ability to objectively confirm MCI or early dementia due to a neurodegenerative disease over either class of measure alone. The current study aimed to examine the extent to which combining data from comprehensive neuropsychological assessment with volumetric assessment of selected mesial temporal subregions on MRI improves the identification of patients with aMCI and mild probable AD dementia in a memory clinic cohort. To address the psychometric limitations associated with isolated neuropsychological test scores, the current study utilized the index scores derived from the Wechsler scales (Wechsler, 2009; Wechsler, 2008), providing a comprehensive evaluation of the principal cognitive constructs defined by the Cattell–Horn–Carroll model of cognition (Agelink van Rentergem et al., 2020; Jewsbury et al., 2017). Furthermore, the current study employed a recently developed automated MRI segmentation method, ASHS‐T1, that has been demonstrated to provide more accurate assessment of the mesial temporal lobe (MTL) subregions (Xie et al., 2019; Yushkevich et al., 2015).

## 2 MATERIALS AND METHODS

### 2.1 Participants

The study sample comprised 40 healthy controls (HCs), 38 patients with subjective memory symptoms (MemS), 16 patients with aMCI, and 16 patients with mild probable AD dementia. The HCs were recruited via advertisements posted throughout St. Vincent's Hospital Melbourne (SVHM) and in community groups within Victoria, Australia. All HCs underwent a comprehensive neuropsychological assessment and a 3.0 T MRI scan. Patients with MemS, aMCI, and mild probable AD dementia were retrospectively identified from archived patient records of the SVHM Cognitive Clinic. These patients were assessed at the SVHM Cognitive Clinic between 2006 and 2018. All patients underwent a neurological assessment, a comprehensive neuropsychological assessment, and either a 1.5 or 3.0 T MRI scan. Clinical diagnosis was made by a neurologist considering the neurological, neuropsychological, and neuroimaging results. Patients with MemS were patients with subjective experience of memory decline but without objective cognitive impairment (i.e., normal performance on standardized cognitive tests, adjusted for age, sex, and education). Patients with aMCI were diagnosed in accordance with the Mayo Clinic (Petersen et al., 1999) or National Institute on Aging–Alzheimer's Association (NIA–AA) (Albert et al., 2011) criteria. Patients with mild probable AD dementia were diagnosed in accordance with the National Institute of Neurological and Communicative Disorders and Stroke–Alzheimer's Disease and Related Disorders Association (NINCDS–ADRDA) (McKhann et al., 1984) or NIA–AA (McKhann et al., 2011) criteria. For all participants, exclusion criteria were: (a) aged below 50 years; and (b) persisting cognitive impairment due to an acquired brain injury. For the HCs, additional exclusion criteria were: (c) current medical or neurological illness that might impact cognition; and (d) abnormal results on neuropsychological assessment or MRI examination. The current study was approved by the SVHM Human Research Ethics Committee (HREC‐A 057/15) and was conducted in accordance with the ethical standards of the Declaration of Helsinki.

### 2.2 Neuropsychological assessment

The neuropsychological assessment included administration of the Wechsler Adult Intelligence Scale (WAIS), the Wechsler Memory Scale (WMS), the Beck Depression Inventory, Second Edition (BDI‐II) (Beck et al., 1996), and the State‐Trait Anxiety Inventory (STAI) (Spielberger et al., 1983). The WAIS and WMS provide broad coverage of core cognitive constructs defined by the Cattell–Horn–Carroll model of cognitive abilities (Agelink van Rentergem et al., 2020; Jewsbury et al., 2017), including so‐called executive function constructs (fluid reasoning, working memory, visual‐spatial processing, and processing speed), with well standardized tests. Given the extended period over which patient data were sampled, some patients were administered the WAIS‐III and WMS‐III, while others were administered the WAIS‐IV and WMS‐IV. Four intelligence measures were calculated from the WAIS subtests: verbal comprehension, perceptual reasoning, working memory, and processing speed. Three memory measures were calculated from the WMS subtests: auditory memory, visual memory, and general memory (mean of Immediate Memory Index and General/Delayed Memory Index from the WMS). The BDI‐II and STAI were used to provide a measure of depression and anxiety symptoms, respectively, to be included as control variables as previous studies have shown that these symptoms are negatively associated with cognitive performance and regional brain volume (Beaudreau & O'Hara, 2009; Faust et al., 2017; O'Shea et al., 2018).

### 2.3 Neuroimaging

#### 2.3.1 MRI acquisition and preprocessing

All T1‐weighted scans were acquired coronally along the hippocampal axis with 3D‐sequenced MPRAGE. 1.5 T scans were acquired using a Siemens Symphony (TR = 1530 ms, TE = 3.41 ms, FOV = 156 × 250 mm^2^, FA = 8°, voxel size = 1.0 × 1.0 × 1.4 mm^3^, matrix = 256 × 160 mm^2^). 3.0 T scans were acquired using a Siemens Skyra (TR = 2200 ms, TE = 2.03 ms, FOV = 230 × 230 mm^2^, FA = 8°, voxel size = 0.9 × 0.9 × 1.0 mm^3^, matrix 256 × 256 mm^2^).

As previously reported (Quek et al., 2023), variations in MRI scan orientation can introduce substantial variability in MRI‐based measurements. Hence, all MRI scans were first segmented using FreeSurfer v6.0.1 and then aligned to a common orientation, perpendicular to the long axis of the FreeSurfer hippocampal segmentation, using a python script (https://doi.org/10.25919/8kjn‐d006), to improve measurement reliability. All MRI scans were then resampled to 0.3 × 0.3 × 1.0 mm^3^ by cubic spline interpolation to enhance scan resolution.

#### 2.3.2 MRI processing

The MTL subregions were automatically segmented using ASHS‐T1 (Xie et al., 2019; Yushkevich et al., 2015). ASHS‐T1 was run using the default parameter settings. Due to differences in the MRI protocol between the ADNI data and the ASHS‐T1 atlas set, the joint label fusion (JLF) output was used (Xie et al., 2019). All segmentations were visually inspected to assess segmentation quality. ASHS‐T1 successfully labeled the MRI scans of all participants. Volume measurements of the entorhinal cortex, transentorhinal cortex, anterior hippocampus, and posterior hippocampus were extracted from the segmentations. Intracranial volume (ICV) was also measured using ASHS‐T1 (Xie et al., 2019).

### 2.4 Statistical analysis

For each brain region, the volume measurements of both hemispheres were summed to yield a total regional volume. The anterior hippocampus and posterior hippocampus volumes were summed to yield a total hippocampus volume. All regional volume measurements were normalized by ICV.

To compare the demographic, psychological, neuropsychological, and MRI variables among the participant groups, one‐way ANOVA or ANCOVA on continuous variables and Pearson's chi‐square test on categorical variables were conducted. The demographic and psychological variables were included as control variables in the ANCOVAs of the neuropsychological and MRI variables. However, only the control variables that significantly contributed to explaining variance in the model were retained. An a priori Bonferroni adjustment was applied to the *p* values to correct for multiple comparisons. Where the tests returned a significant result, Bonferroni‐adjusted post hoc comparisons were undertaken to investigate pairwise differences. Effect sizes, eta‐squared and partial eta‐squared for one‐way ANOVA and ANCOVA, respectively, and Cramer's *V* for Pearson's chi‐square test, were also calculated. Eta‐squared and partial eta‐squared were interpreted as trivial (< 0.01), small (0.01−0.06), medium (0.06−0.14), or large (≥ 0.14) (Cohen, 1988). Interpretation of Cramer's *V* was corrected for the number of degrees of freedom (Cohen, 1988).

To evaluate the utility of the neuropsychological and MRI variables in predicting group membership, multinomial logistic regression was employed. To facilitate comparison among the different classes of measures (i.e., neuropsychological and MRI measures), the raw scores of the predictor variables were converted to *z*‐scores using the overall group mean and standard deviation. Separate neuropsychological and MRI measures models were constructed using the backward stepwise variable selection method, with variable inclusion determined using a likelihood‐ratio test with entry probability set at *p* < .15 and removal probability set at *p* < .20. The backward stepwise method was selected as it is more likely to select stronger models and is more robust to high collinearity among the predictor variables (Royston & Sauerbrei, 2008). For the memory measures, only general memory was included in the model as both auditory and visual memory are measured within general memory (Holdnack et al., 2011; Price et al., 2002). The variables included in the MRI measures model were then added to the variables included in the neuropsychological measures model to derive a combined measures model. An a priori Bonferroni adjustment was applied to the *p* values of the pairwise comparisons within each model to correct for multiple comparisons. The neuropsychological and MRI measures models were compared to the combined measures model using the likelihood‐ratio test to examine the relative contribution of each model to the prediction of group membership.

## 3 RESULTS

### 3.1 Demographic, psychological, neuropsychological, and MRI variables

The sample demographic, psychological, neuropsychological, and MRI variables are shown in Table 1. The HC group scored significantly higher on working memory, processing speed, auditory memory, and general memory compared to the MemS group and significantly higher on all intelligence and memory measures compared to the MCI and AD groups. The MemS group scored significantly higher on processing speed and all memory measures compared to the MCI group and significantly higher on all intelligence and memory measures compared to the AD group. There were no significant differences among the groups in entorhinal cortex volume. The HC group showed significantly larger transentorhinal cortex and hippocampal volumes compared to the aMCI and AD groups. The MemS group showed significantly larger transentorhinal cortex and hippocampal volumes compared to the AD group.

**TABLE 1:** Sample demographic, psychological, neuropsychological, and MRI variables.

Characteristic	HC (*n* = 40)	MemS (*n* = 38)	aMCI (*n* = 16)	AD (*n* = 16)	Covariates^a^	*p* Value^b^	Effect size^c^	Pairwise comparisons
**Demographic variables**								
Age (years)	67.75 ± 5.41	66.16 ± 9.38	72.19 ± 7.48	72.38 ± 8.43	–	.011	0.10	–
Gender (F:M %)	75.0:25.0	57.9:42.1	37.5:62.5	37.5:62.5	–	.016	0.31	–
Education (years)	15.95 ± 2.68	13.26 ± 4.15	11.31 ± 3.22	12.13 ± 4.59	–	3.5E‐5*	0.20	HC > MemS, aMCI, AD
**Psychological variables**								
Depression symptoms	4.43 ± 4.11	11.83 ± 9.91	11.00 ± 6.25	9.57 ± 8.48	–	3.8E‐4*	0.20	HC < MemS
State anxiety symptoms	31.58 ± 8.60	38.15 ± 15.51	40.30 ± 8.99	36.81 ± 8.84	–	.053	0.08	–
Trait anxiety symptoms	33.13 ± 7.26	41.79 ± 14.68	39.60 ± 7.46	39.75 ± 9.16	–	.008	0.13	–
**Neuropsychological variables**								
Verbal comprehension	118.95 ± 12.07	107.11 ± 15.02	97.00 ± 12.44	94.00 ± 17.39	Age, Edu	5.0E‐6*	0.23	HC > aMCI, AD; MemS > AD
Perceptual reasoning	115.35 ± 11.56	103.39 ± 17.39	93.31 ± 12.67	86.63 ± 19.77	Edu	9.0E‐6*	0.22	HC > aMCI, AD; MemS > AD
Working memory	114.98 ± 11.91	101.26 ± 15.50	95.81 ± 9.69	87.38 ± 15.51	Edu	7.9E‐7*	0.26	HC > MemS, aMCI, AD; MemS > AD
Processing speed	114.70 ± 9.98	98.13 ± 11.72	87.50 ± 8.74	82.87 ± 15.59	Edu	1.9E‐13*	0.45	HC > MemS, aMCI, AD; MemS > aMCI, AD
Auditory memory	116.95 ± 12.36	99.33 ± 16.67	76.72 ± 13.60	66.25 ± 12.98	–	3.2E‐23*	0.64	HC > MemS, aMCI, AD; MemS > aMCI, AD
Visual memory	106.68 ± 18.07	99.83 ± 13.57	84.06 ± 14.52	73.34 ± 10.03	–	1.9E‐11*	0.39	HC > aMCI, AD; MemS > aMCI, AD
General memory	115.64 ± 10.77	99.66 ± 15.17	77.13 ± 13.05	65.63 ± 10.41	–	5.8E‐26*	0.68	HC > MemS, aMCI, AD; MemS > aMCI, AD
**MRI variables**								
Entorhinal cortex volume (mm^3^)	949.07 ± 116.45	964.35 ± 142.06	880.58 ± 205.28	834.85 ± 149.01	–	.011	0.10	–
Transentorhinal cortex volume (mm^3^)	1046.07 ± 161.42	984.32 ± 169.92	891.19 ± 241.79	823.28 ± 155.04	–	1.1E‐4*	0.18	HC > aMCI, AD; MemS > AD
Hippocampal volume (mm^3^)	6707.54 ± 692.46	6466.13 ± 947.16	6137.54 ± 1056.43	5590.83 ± 716.31	–	3.4E‐7*	0.27	HC > aMCI, AD; MemS > AD

*Note*: For the MRI variables, data are presented as raw values, but the between‐group statistical analyses were conducted on the ICV‐normalized values.

^a^ Demographic and psychological variables were included as control variables in the ANCOVAs of the neuropsychological and MRI variables. In these analyses, only statistically significant control variables were retained.

^b^ For continuous variables, one‐way ANOVA or ANCOVA was conducted. For categorical variables, Pearson's chi‐square test was conducted.

^c^ Effect sizes are η^2^ for one‐way ANOVAs, η_p_2 for one‐way ANCOVAs, and Cramer's *V* for Pearson's chi‐square tests.

*p < .05/16 = .003 (Bonferroni‐adjusted significance level).

AD = Alzheimer's disease; aMCI = amnestic mild cognitive impairment; HC = healthy control; MemS = memory symptoms; MRI = magnetic resonance imaging.

### 3.2 Predicting group membership

**FIGURE 1:**
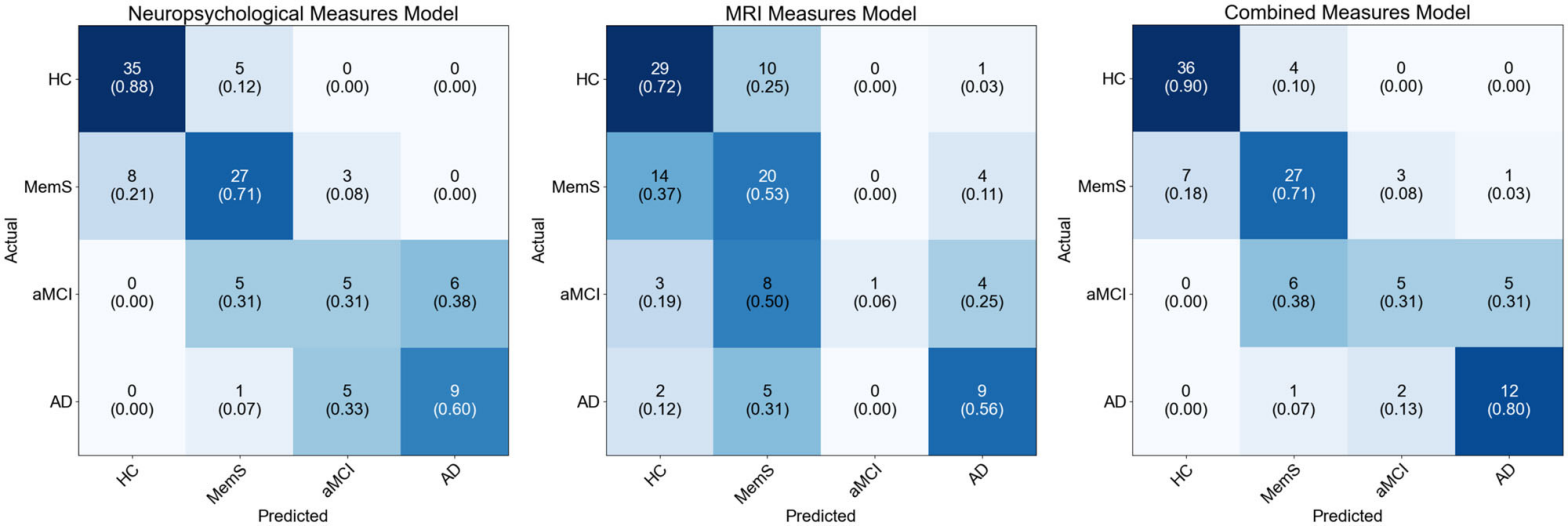
Confusion matrices of the multinomial logistic regression models. The rows represent the actual groups while the columns represent the predicted groups. The diagonal cells represent the correctly predicted cases, and the off‐diagonal cells represent the mis‐predicted cases. Shading represents the predicted case count, with darker shading representing higher predicted case count. AD = Alzheimer's disease; aMCI = amnestic mild cognitive impairment; HC = healthy control; MemS = memory symptoms; MRI = magnetic resonance imaging.

As the demographic and psychological variables were not consistently found to be significant control variables in the ANCOVAs of the neuropsychological and MRI variables, these variables were not included as control variables in the subsequent multinomial logistic regression analyses. The multinomial logistic regression models predicting group membership are summarized in Table 2. The classification results of the multinomial logistic regression models are presented in Figure 1.

**TABLE 2:** Multinomial logistic regression models predicting group membership.

Model, variable	Model fit		HC vs. MemS			HC vs. aMCI			HC vs. AD		
	−2 Log likelihood	Nagelkerke *R* ^2^	*B*	*p* Value	OR (95% CI)	*B*	*p* Value	OR (95% CI)	*B*	*p* Value	OR (95% CI)
**Neuropsychological measures**	142.01	0.78									
Processing speed			−2.22	1.2E‐4*	0.11 (0.04−0.34)	−3.21	1.3E‐4*	0.04 (0.01−0.21)	−3.27	3.9E‐4*	0.04 (0.01−0.23)
General memory			−1.79	.004*	0.17 (0.05−0.57)	−4.33	2.1E‐5*	0.01 (0.002−0.10)	−6.36	1.5E‐6*	0.002 (0.0001−0.02)
**MRI measures**	239.33	0.37									
Entorhinal cortex volume			1.22	.010*	3.26 (1.38−7.70)	0.97	.103	2.03 (0.69−5.91)	1.60	.007*	2.66 (0.90−7.86)
Transentorhinal cortex volume			−0.86	.025	0.42 (0.20−0.85)	−1.21	.018	0.32 (0.13−0.82)	−1.24	.025	0.30 (0.11−0.82)
Hippocampal volume			−0.87	.025	0.45 (0.22−0.94)	−1.12	.028	0.49 (0.19−1.23)	−2.43	1.3E‐4*	0.20 (0.07−0.59)
**Neuropsychological + MRI measures**	129.75	0.81									
Processing speed			−2.25	2.9E‐4*	0.11 (0.03−0.36)	−3.59	1.7E‐4*	0.03 (0.004−0.18)	−3.72	4.4E‐4*	0.02 (0.003−0.19)
General memory			−1.77	.007*	0.17 (0.05−0.62)	−4.50	6.8E‐5*	0.01 (0.001−0.10)	−6.88	8.3E‐6*	0.001 (0.00005−0.02)
Entorhinal cortex volume			0.61	.326	1.84 (0.55−6.21)	0.25	.792	1.29 (0.20−8.47)	0.50	.627	1.65 (0.22−12.44)
Transentorhinal cortex volume			−0.96	.094	0.38 (0.13−1.18)	−1.83	.039	0.16 (0.03−0.91)	−1.47	.139	0.23 (0.03−1.61)
Hippocampal volume			−0.07	.892	0.93 (0.33−2.62)	0.69	.439	1.98 (0.35−11.23)	−0.59	.574	0.55 (0.07−4.37)

AD = Alzheimer's disease; aMCI = amnestic mild cognitive impairment; HC = healthy control; MemS = memory symptoms; MRI = magnetic resonance imaging.

*p < .05/3 = .017 (Bonferroni‐adjusted significance level).

#### 3.2.1 Neuropsychological measures model

To select the neuropsychological variables for inclusion in the neuropsychological measures model, a backward stepwise multinomial logistic regression analysis was used. Although all neuropsychological variables were entered into the analysis, only processing speed and general memory emerged as significant predictors of group membership. Hence, the neuropsychological measures model comprised processing speed and general memory.

The neuropsychological measures model significantly predicted group membership, χ^2^(6) = 139.18, *p* < .001, Nagelkerke *R* ^2^ = 0.78. Both processing speed and general memory discriminated MemS, aMCI, and AD from HC. The overall accuracy of the neuropsychological measures model was 0.70 (95% CI 0.60 to 0.78). The accuracy of the neuropsychological measures model for classifying HC, MemS, aMCI, and AD was 0.88 (95% CI 0.73 to 0.96), 0.71 (95% CI 0.54 to 0.85), 0.31 (95% CI 0.11 to 0.59), and 0.60 (95% CI 0.32 to 0.84), respectively. However, the 95% CIs of the accuracy for classifying aMCI and AD included 0.50, indicating that the neuropsychological measures model did not perform significantly better than chance at classifying these groups.

#### 3.2.2 MRI measures model

To select the MRI variables for inclusion in the MRI measures model, a backward stepwise multinomial logistic regression analysis was used. Entorhinal cortex volume transentorhinal cortex volume and hippocampal volume all emerged as significant predictors of group membership. Hence, the MRI measures model comprised entorhinal cortex volume, transentorhinal cortex volume, and hippocampal volume.

The MRI measures model significantly predicted group membership, χ^2^(9) = 45.76, *p* < .001, Nagelkerke *R* ^2^ = 0.37. Entorhinal cortex volume discriminated MemS and AD from HC but did not discriminate aMCI from HC. Transentorhinal cortex volume did not discriminate MemS, aMCI, or AD from HC. Hippocampal volume discriminated AD from HC but did not discriminate MemS or aMCI from HC. The overall accuracy of the MRI measures model was 0.54 (95% CI 0.44 to 0.63). The accuracy of the MRI measures model for classifying HC, MemS, aMCI, and AD was 0.73 (95% CI 0.56 to 0.85), 0.53 (95% CI 0.36 to 0.69), 0.06 (95% CI 0.00 to 0.30), and 0.56 (95% CI 0.30 to 0.80), respectively. However, the 95% CIs of the overall accuracy and of the accuracy for classifying HC, MemS, aMCI, and AD either included or were less than 0.50, indicating that the MRI measures model did not perform significantly better than chance at classifying these groups.

#### 3.2.3 Neuropsychological + MRI measures model

The variables included in both the neuropsychological measures model and the MRI measures model were combined to create a combined neuropsychological and MRI measures model. Hence, the combined measures model comprised processing speed, general memory, entorhinal cortex volume, transentorhinal cortex volume, and hippocampal volume.

The combined neuropsychological and MRI measures model significantly predicted group membership, χ^2^(15) = 151.43, *p* < .001, Nagelkerke *R* ^2^ = 0.81. Processing speed and general memory, but not hippocampal volume, entorhinal cortex volume, or transentorhinal cortex volume, significantly predicted group membership. Both processing speed and general memory discriminated MemS, aMCI, and AD from HC. Entorhinal cortex volume, transentorhinal cortex volume, and hippocampal volume did not discriminate MemS, aMCI, or AD from HC. The overall accuracy of the combined neuropsychological and MRI measures model was 0.73 (95% CI 0.64 to 0.81). The accuracy of the combined neuropsychological and MRI measures model for classifying HC, MemS, aMCI, and AD was 0.90 (95% CI 0.76 to 0.97), 0.71 (95% CI 0.54 to 0.85), 0.31 (95% CI 0.11 to 0.59), and 0.80 (95% CI 0.52 to 0.96), respectively. However, the 95% CIs of the accuracy for classifying aMCI included 0.50, indicating that the combined neuropsychological and MRI measures model did not perform significantly better than chance at classifying the aMCI group.

Likelihood‐ratio tests comparing the neuropsychological and MRI measures models to the combined neuropsychological and MRI measures model indicated that the combined measures model did not significantly improve group membership prediction over the neuropsychological measures model, χ^2^(9) = 12.26, *p* = .199 but did significantly improve group membership prediction over the MRI measures model, χ^2^(6) = 107.44, *p* = 7.0E‐21.

#### 3.2.4 Supplementary analyses: exclusion of entorhinal cortex volume

Given the absence of a statistically significant difference in entorhinal cortex volume among the participant groups (see Table 1), the multinomial logistic regression analyses were rerun with entorhinal cortex volume excluded to examine its impact on the statistical results. Exclusion of entorhinal cortex volume did not alter the pattern of statistical significance of the results of the multinomial logistic regression analyses.

## 4 DISCUSSION

The current study sought to investigate the utility of combining neuropsychological and MRI measures to clinically group patients, particularly those with aMCI, in a memory clinic cohort. General memory and processing speed were identified as the neuropsychological measures that contributed most to predicting group membership. All MRI measures, namely, entorhinal cortex volume, transentorhinal cortex volume, and hippocampal volume, were found to be important predictors of group membership. The neuropsychological measures showed higher overall classification accuracy compared to the MRI measures, and, importantly, the addition of the MRI measures to the neuropsychological measures did not improve group membership prediction.

### 4.1 Neuropsychological measures

Among the neuropsychological measures, general memory and processing speed emerged as the most important predictors to discriminate between HCs, patients with MemS, patients with aMCI, and patients with mild probable AD dementia. The combination of these measures achieved an overall 70% accuracy at predicting group membership. These measures, however, performed poorly at specifically identifying patients with aMCI, with accuracy only at 31%. The confusion matrix of the neuropsychological measures model indicated that these measures misclassified more than one‐third of the patients with aMCI as being patients with mild probable AD dementia. Indeed, in the group mean comparisons, neither general memory nor processing speed, nor any of the other neuropsychological measures, were significantly different between the aMCI and AD groups. These findings are in contrast to previous work showing that patients with AD dementia perform worse on memory, language, processing speed, working memory, and visuospatial tasks compared to patients with aMCI (Carter et al., 2012; Economou et al., 2007; Hildebrandt et al., 2013). In the current study, the absence of any difference in the neuropsychological measures between these two patient groups is likely because these groups were inadequately sized for the detection of small but significant group differences. Importantly, although the neuropsychological measures performed poorly at discriminating between patients with aMCI and patients with mild probable AD dementia, these measures, nevertheless, performed well at generally identifying patients likely to have AD. Overall, these findings support the use of neuropsychological measures in the diagnosis of AD but also highlight the need for further evaluation of these measures.

### 4.2 MRI measures

Among the MRI measures, all measures, namely, entorhinal cortex volume, transentorhinal cortex volume, and hippocampal volume, emerged as important predictors to discriminate between HCs, patients with MemS, patients with aMCI, and patients with mild probable AD dementia. However, the overall accuracy of these measures at predicting group membership was only 54%, and the accuracy to identify patients with aMCI was 6%. The overall poor performance of the MRI measures is unexpected, given consistent findings of entorhinal cortex, transentorhinal cortex, and hippocampal atrophy in patients with aMCI and patients with AD dementia relative to HCs (Du et al., 2001; Pennanen et al., 2004; Xie et al., 2019). One explanation for the poor performance of the MRI measures is that the method used to derive the MRI measures, ASHS‐T1, is not sufficiently robust to detect disease‐related differences between the participant groups. Supporting such an interpretation, the group mean comparisons did not identify a difference in entorhinal cortex volume between the HC and aMCI groups or in any of the regional volumes between the aMCI and AD groups. While ASHS‐T1 has previously demonstrated sensitivity to differences in the volumes of the mesial temporal lobe subregions across the stages of AD, these results, to our knowledge, have only been examined on MRI scans that are acquired according to highly standardized protocols and meet rigorous quality control standards (Xie et al., 2019; Xie et al., 2017; Xie et al., 2016). The current study, in contrast, utilized MRI scans that were acquired in a clinical environment and not subject to similarly strict standards. Hence, the performance of ASHS‐T1 may have been influenced by variations in image quality (e.g., signal‐ and contrast‐to‐noise ratio) or by the presence of image artifacts, to the extent that disease‐related differences were masked (Kruggel et al., 2010; Reuter et al., 2015). Evaluation of the impact of image quality and artifacts on ASHS‐T1 measurements would assist in further understanding the capabilities and limitations of the segmentation method.

### 4.3 Combination of neuropsychological and MRI measures

The addition of the MRI measures to the neuropsychological measures did not improve the prediction of group membership, suggesting that the MRI measures contributed no diagnostic utility over and above the neuropsychological measures. Two other studies have reported on the combined use of neuropsychological and MRI measures to identify individuals with AD. These studies, using a different combination of neuropsychological and MRI measures, likewise found nonsignificant improvements in diagnostic accuracy offered by the addition of the MRI measures to the neuropsychological measures (Goryawala et al., 2015; Liu et al., 2011). The consistency of findings of the relative unimportance of MRI measures over neuropsychological measures might suggest that MRI measures yield no unique contribution to the diagnosis of AD when neuropsychological measures have been taken into account. Such an interpretation would imply that neuropsychological and MRI measures are essentially measures of the same disease process, with neuropsychological measures being the superior marker. While there is indeed a close association between neuropsychological and MRI measures in patients with AD, there is still substantial unexplained variance in the association, indicating that neuropsychological and MRI measures are measuring related yet distinct disease processes (Dickerson et al., 2009; McDonald et al., 2012). Alternatively, as previously proposed, the poor performance of MRI measures may be a reflection of the methods used to obtain these data. Previous studies have shown that the performance of automated MRI segmentation methods may be influenced by numerous factors, such as acquisition parameters, head positioning, software variables, and structure characteristics (Haller et al., 2016; Hedges et al., 2022; Morey et al., 2010). Concerningly, the degree of variability in the measurements derived from automated segmentation methods as a result of these factors can be sufficiently large as to obscure important between‐groups differences in early AD (Haller et al., 2016). While the generalizability of these results to other automated segmentation methods remains to be studied, these findings suggest that automated MRI segmentation may not be sufficiently developed to provide independent clinical utility.

### 4.4 Limitations

There are several limitations to the current study that warrant consideration. First, the sample sizes of the aMCI and AD groups were arguably limited. Second, the clinical diagnoses of aMCI and mild probable AD dementia were not supplemented by disease‐specific AD biomarkers, such as amyloid PET imaging. Nevertheless, a counterargument to these limitations is that the patients in the current study were very well characterized by a comprehensive clinical workup, which included neurological, neuropsychological, and neuroimaging assessments. Third, and relatedly, patient diagnoses were formulated based, in part, on the neuropsychological assessment results and a qualitative evaluation of the MRI scans. Consequently, the inclusion of neuropsychological test scores and MRI‐based regional brain volumes as predictor variables in the logistic regression models may have resulted in an overestimation of the diagnostic accuracy statistics. Nevertheless, the primary objective of the current study was not to quantify the diagnostic accuracy of the neuropsychological and MRI measures but rather to investigate the relative contribution of each to the overall diagnostic accuracy despite the potential bias inherent in clinicians’ knowledge of prior investigation results. Ideally, a future study would derive diagnostic accuracy rates that are blinded to the results of the clinical investigation. Fourth, due to the extended period over which patient data were sampled, the data comprised some patients who were administered the third edition of the WAIS and WMS and some patients who were administered the fourth edition of the WAIS and WMS. Nevertheless, comparisons of the third and fourth editions of the WAIS and WMS have shown high correlations (*r* > 0.80) between corresponding index scores (Wechsler, 2009; Wechsler, 2008), suggesting that corresponding index scores measure similar constructs. Accordingly, the use of the third and fourth editions of the WAIS and WMS in the current study is unlikely to have had a significant impact on the findings.

## 5 CONCLUSION

Overall, the findings of the current study suggest that MRI‐based measures contribute little to the detection of early AD over and above reliable neuropsychological measures. While automated MRI segmentation remains a promising avenue to bring objective and timely assessment of brain atrophy to routine clinical workup, further refinement of segmentation methods is required in order for it to achieve more robust diagnostic utility. Consequently, it may be argued that comprehensive neuropsychological assessment remains a priority in the workup of patients with suspected early AD.

## AUTHOR CONTRIBUTIONS

**Yi‐En Quek**: Conceptualization; data curation; formal analysis; investigation; methodology; project administration; software; visualization; writing—original draft; writing—review and editing. **Yi Leng Fung**: Conceptualization; data curation; funding acquisition; investigation; resources; writing—review and editing. **Pierrick Bourgeat**: Resources; software; writing—review and editing. **Simon J. Vogrin**: Data curation; investigation; resources; writing—review and editing. **Steven J. Collins**: Data curation; investigation; resources; supervision; writing—review and editing. **Stephen C. Bowden**: Funding acquisition; resources; supervision; writing—review and editing.

## CONFLICT OF INTEREST STATEMENT

Portions of these findings were presented as a poster at the Alzheimer's Association International Conference 2023, Amsterdam, Netherlands. The authors declare no conflicts of interest.

### PEER REVIEW

The peer review history for this article is available at https://publons.com/publon/10.1002/brb3.3505.

## Data Availability

The data that support the findings of this study are available on request from the corresponding author. The data are not publicly available due to privacy or ethical restrictions.
